# Battles of the Soul: Validation of the Scale of Religious and Spiritual Struggles (RSS) for the Portuguese Population

**DOI:** 10.1007/s10943-023-01953-x

**Published:** 2023-12-06

**Authors:** Carla Tomás, Ana Moreira

**Affiliations:** 1 Department of Psychology and Sports, Instituto Superior Manuel Teixeira Gomes, 8500-590 Portimão, Portugal; 2 School of Psychology, ISPA—Instituto Universitário, Rua do Jardim do Tabaco 34, 1149-041 Lisbon, Portugal; 3 APPsyCI—Applied Psychology Research Center Capabilities & Inclusion, ISPA—Instituto Universitário, R. Jardim do Tabaco 34, 1149-041 Lisbon, Portugal; 4 Faculdade de Ciências e Tecnologia, Universidade Europeia, Quinta do Bom Nome, Estr. da Correia 53, 1500-210 Lisbon, Portugal

**Keywords:** Religion, Psychology, Spirituality, Struggles, Quantitative study

## Abstract

This study aims to adapt and validate the Religious and Spiritual Struggles Scale for the Portuguese population. The sample consisted of 732 participants with various religious affiliations. The exploratory factor analysis showed that it consists of six dimensions, similar to the initial instrument. A KMO of 0.91 was obtained. The confirmatory factor analysis confirmed the existence of six factors and showed adequate fit indices. Internal consistency and construct reliability were above 0.70. The analysis of the psychometric qualities of this instrument indicates that it can be applied to the Portuguese population and is a valuable instrument for psychotherapeutic practice and studies in the psychology of religion and spirituality.

## Introduction

Human existence is touched by moments of doubt and emotional suffering in the relationship that individuals establish with the Sacred. We call these phenomena religious/spiritual struggles (RSS). These battles often arise in response to overwhelming events that call into question the worldview (and the system of spiritual orientation) that has previously guided a person in the adaptation to life events.

These battles, which involve life circumstances that the individual associates with the Transcendent, are common and can be found in people of any gender, religious, age, social, cultural, or ethnic group (Abu-Raiya et al., 2015a, 2015b).

Due to the frequency, scope and consequences for health and well-being of RSS, it is essential to identify and intervene in these occurrences. With this aim in mind and because there is currently no instrument in Portugal to measure the presence and intensity of RSS, the Religious and Spiritual Struggles Scale (Exline et al., 2014) was validated for a European Portuguese population.

## Literature Review

### Religious and Spiritual Struggles

Religious and spiritual struggles manifest as a state of tension, conflict, and doubt that focuses on issues of faith, the relationship with the Divine and relationships within and between religious communities (Exline, 2013; Exline & Rose, 2014; Pargament & Exline, 2022). These are sometimes one-off events, but for some people, they are circumstances that are faced repeatedly.

The terrain on which these battles take place can be diverse and may include the individual, others and the Supernatural. Furthermore, the nature and location of these clashes define the form that RSS take. According to Pargament and Exline (2022), RSS can be classified into three broad categories: supernatural struggles focus on the discomfort brought by unfavourable perceptions of spiritual entities, which can be Divine (God or Gods) or demonic, evil spirits; intrapsychic struggles refer to internal conflicts based on cognitions about the observance of moral norms, the search for an ultimate purpose and questioning faith; and interpersonal struggles occur in the presence of tensions with other people who share the same faith or between subjects with different spiritual beliefs. Within these more general categories, we identify six types of specific RSS, which are described in Table 1 (Exline & Rose, 2014; Exline et al., 2014; Pargament & Exline, 2022):

**Table 1:** Typology of spiritual struggles

Supernatural struggles	*Divine Struggles*: One of the most studied types of RS tension, it evokes issues, conflicts and tensions associated with the image of God and the individual’s perception of the quality of the relationship established with the Divine. Feelings of disappointment, anger, punishment, abandonment, or lack of love from God are common in these types of struggles
	*Demonic struggles*: These arise when individuals consider the intervention of the Devil or evil spirits as the cause of their personal problems and feel attacked or tormented by these types of supernatural forces
Intrapersonal struggles	*Struggles of doubt*: Happen in moments of uncertainty and confusion about RS beliefs, and can take different forms, from questioning broader issues of faith, such as the existence of God, or just scepticism about more concrete aspects of RS experience, such as the position of a particular religious community concerning a specific issue of RS practice
	*Moral struggles*: Occur in the presence of inconsistencies between the values defended by the individual and the actions practiced, manifesting themselves through tension, guilt, awareness of imperfection, and the inability to live up to higher standards
	*Struggles for ultimate meaning*: These occur in the presence of uncertainty about the ultimate purpose of life, either because it has not yet been found or because of the need to redefine a new meaning when the previous one is no longer coherent
Interpersonal struggles	*Interpersonal struggles*: These are one of the most common types of struggles and occur in the presence of fractious issues in interpersonal relationships, or in the judgement that is made about how faith communities reflect in their daily actions the doctrine of faith they profess. These disagreements can occur between people from the same congregation or different RS faiths

RSS are multicausal; they may appear due to internal and external events in subjects’ lives and shake up individual beliefs, either because of the immediate intensity of the suffering they cause or because of their cumulative nature (Ano & Pargament, 2013). Examples of these events include the onset of a severe and life-threatening illness (Fitchett et al., 2004; Magyar-Russell et al., 2014; Park et al., 2009, 2011), mental illness (Exline et al., 2000; Murphy et al., 2016; Zarzycka & Zietek, 2019), bereavement (Burke et al., 2014; Cowchock et al., 2010), or acute stress situations (Currier et al., 2018), such as sexual abuse or exposure to a natural disaster.

These events shake the spiritual guidance system (SOS) of individuals who tend to create assumptions of the world as fundamentally good and just (Pargament, 1997) and generate incomprehensibility and pain in the face of experiences of inequity and suffering. The SOS functions as a comprehensive lens through which individuals interpret their experiences in the world. It can be a determining factor in both the onset and the outcome of religious and spiritual struggles according to four of its characteristics, which can function as facilitators or barriers in the adjustment to existential challenges (Pargament et al., 2006):

- spiritual integration (the degree of congruence between religious/spiritual beliefs and practices in everyday life);
- flexibility (religious/spiritual plasticity, especially in the presence of unexpected events, and the varied repertoire of responses generated according to the characteristics of the precipitating event);
- differentiation (the ability to appreciate the paradox of life events, even religious and spiritual events, and avoid oversimplification of events and ideas); and
- benevolence (the intensity of kindness and comfort perceived and experienced with the Divine and the religious/spiritual community).

Exposure to these religious and spiritual stresses does not leave individuals undamaged. Although their effects can manifest in different ways, they usually induce suffering and put constraints on health and personal well-being (Ano & Vasconcelles, 2005; Bockrath et al., 2022; Exline, 2013; Exline & Rose, 2014; Pargament, 2007; Reynolds et al., 2016; Sedlar et al., 2018). The harmful consequences that remain after RSS include depression (Abu-Raiya et al., 2016b), anxiety (Exline, 2013), suicidal ideation (Rosmarin et al., 2013), stress (Ellison & Lee, 2010), lower levels of life satisfaction (Abu-Raiya et al., 2015a, 2016a), lower well-being (Wilt et al., 2017), symptoms of physical pain and a higher risk of mortality (Pargament et al., 2001).

Religious and spiritual struggles can be short-lived or prolonged in time, and their manifestation can vary in intensity. For most people, they bring moments of anguish and disturbance (Pargament & Exline, 2022). Given the robust links demonstrated in the literature between RSS and their negative impact on health and well-being indices (Exline, 2013; Exline & Rose, 2014; Exline et al., 2014; Sedlar et al., 2018), it would be inappropriate to ignore them in any psychological assessment and intervention (Pargament, 2007; Pargament & Exline, 2022). This is why the availability of reliable measuring instruments for this reality is essential.

In addition, it should be kept in mind that RSS do not always have a negative outcome. In some circumstances, they can lead to psychological growth and more mature faith and may promote personal development benefits due to psychological measures that make it possible to monitor and understand the process of spiritual struggles.

### Religious and Spiritual Struggles (RSS) Scale

Faced with the lack of an instrument to measure the various dimensions of RSS in an exclusive but comprehensive way, Exline et al. (2014) created the Religious and Spiritual Struggles (RSS) scale, which reliably, concisely and flexibly addresses the subjective experience of RSS and can be used both with individuals who believe in the extraordinary and with those who have a more atheistic view of the world.

Assessing RSS through the RSS scale provides several benefits to understand these episodes, particularly from a methodological point of view. For example, it provides the possibility of understanding RSS with just one instrument since religious/spiritual-based tensions have previously been assessed in a dispersed and limited way by measures that were not specific to this variable. An example of these scales is the RCOPE (Pargament et al., 2000), which includes a few items that measure divine struggles, diabolical struggles, and interpersonal struggles. This scale, which measures RS coping, has been validated for the Portuguese population in a longer version (Tomás & Rosa, 2021) and in the Brief RCOPE version (Casaleiro et al., 2022).

Studies have shown that the RSS scale has good psychometric qualities, making it possible to quickly and effectively assess conflicts associated with faith experiences (Exline et al., 2014). This scale has since been validated for other linguistic and cultural contexts, such as Brazil (Esperandio et al., 2022), the Czech Republic (Janů et al., 2018), Poland (Zarzycka et al., 2018), Israel (Abu-Raiya et al., 2015b, 2016a). Most studies have involved Christian samples, but the RSS has also been adapted to people of Muslim faith (Abu-Raiya et al., 2015b), Jewish faith (Abu-Raiya et al., 2016a), and individuals who declared themselves atheists (Sedlar et al., 2018) or nonreligious (Stauner et al., 2016). More recently, a shorter version of the scale was created (Exline et al., 2022) with only 14 items (RSS-14), all taken from the longer scale, to make the instrument more practical and attractive. The reduced scale has been validated for Polish (Falewicz et al., 2023) and Brazilian (Esperandio et al., 2022) populations.

## Method

### Data Collection Procedure

The translation and cultural adaptation of the original language (English) into European Portuguese was carried out after authorization by the author of the RSS scale. The Portuguese-language version of the Religious and Spiritual Struggles Scale used in this study was translated from its English version by two professionals in the field of psychology with mastery of the subject and a high level of knowledge of the English language. The authors of this study standardized the translations. The back-translation work was carried out by two Portuguese professionals with extensive knowledge of English. The authors of this study carried out the convergence of the back-translated versions with the originals in English.

The sampling process was nonprobabilistic, convenience, intentional snowball sampling (Trochim, 2000). The questionnaire, which was posted online on the Google Forms platform, contained information about the purpose of the study and informed consent and guaranteed the confidentiality of the data. After reading the informed consent form, the participants answered a question about whether they agreed to answer the questionnaire. If they answered no, they were taken to the end of the questionnaire.

The questionnaire was posted on social media (Facebook and LinkedIn), and data collection occurred between August 2022 and September 2023.

### Participants

This study’s sample consisted of 732 participants of Portuguese nationality aged 16 to 76 (M = 43.26; SD = 14.35). Among the participants, 530 (72.4%) were female and 202 (27.6%) were male. Concerning educational level, 13 (1.8%) had primary education, 162 (22.1%) had secondary education, 354 (48.4%) had a bachelor’s degree, 169 (23.1%) had a master’s degree, and 34 (4.6%) had a doctorate. In terms of occupational status, 101 (13.8%) were students, 552 (74.5%) were workers, 33 (4.5%) were unemployed, and 46 (6.3%) were retired. For marital status, 285 (35.2%) were single, 384 (52.5%) were married or in a civil partnership, 68 (9.3%) were divorced, and 22 (3%) were widowed. Among these participants, 538 (73.5%) were Catholics, 24 (3.3%) were Protestants, 35 (4.8%) were Evangelicals, 1 (0.15%) was Muslim, 2 (0.3%) were Jewish, 20 (2.7%) were followers of another religion, and 112 (15.3%) did not consider themselves religious.

### Materials

The instrument to be adapted for the Portuguese population was the Religious and Spiritual Struggles Scale developed by Exline et al. (2014). This instrument consists of 26 items with five response options: not at all (1), a little bit (2), somewhat (3), quite a bit (4) and a great deal (5). The 26 items are divided into six dimensions: divine struggles (Items 2, 9, 16, 19 and 24), demonic struggles (Items 6, 11, 18 and 25), struggles with doubt (Items 5, 15, 20 and 23), moral struggles (Items 1, 8, 14 and 21), struggles with ultimate meaning/purpose (Items 3, 7, 12 and 22), and spiritual struggles (4, 10, 13, 17 and 26). There are no reversed items. The scale can be completed in reference to a specific time point (e.g., the past week or month) or a specific event (e.g., a health crisis or a loss).

### Data Analysis Procedure

The first step was to import the data into SPSS Statistics 29 software (IBM Corp., Armonk, NY., USA). We followed the procedures of Koenig and Al Zaben (2021) to adapt this instrument to the Portuguese population. The sample was then randomly divided into two parts, each with 366 participants. An exploratory factor analysis was conducted on one of the parts. The KMO value was calculated, which should be greater than 0.70 (Sharma, 1996). We also calculated the average variance extracted, which should be greater than 50%. For the factor weights of each item, all items with factor weights greater than 0.50 were considered. Internal consistency was tested for each of the dimensions of the instrument by calculating Cronbach’s alpha, which must be greater than 0.70 (Bryman & Cramer, 2003).

Two confirmatory factor analyses were carried out with the other part of the sample, one factor and six factors. The confirmatory factor analyses were conducted using AMOS Graphics software for Windows (IBM Corp., Armonk, NY, USA). The procedure followed a “model generation” logic (Jöreskog & Sörbom, 1993). Following the established recommendations (Hu & Bentler, 1999), six fit indices were combined: chi-square ratio/degrees of freedom (χ^2^/gl); Tucker‒Lewis index (TLI); goodness-of-fit index (GFI); comparative fit index (CFI); root mean square error of approximation (RMSEA); and root mean square residual (RMSR). The chi-square/degrees of freedom ratio (χ^2^/gl) is considered acceptable if it is below 5. For the CFI, GFI and TLI, values above 0.90 indicate a good fit and values between 0.80 and 0.90 indicate an acceptable fit. For RMSEA, values below 0.08 indicate a good fit (MacCallum et al., 1996). The lower the RMSR is, the better the fit (Hu & Bentler, 1999). We tested the construct reliability for each scale’s dimensions, which should be greater than 0.70. Finally, convergent validity was tested by calculating the average variance extracted (AVE), which should be greater than 0.50 (Fornell & Larcker, 1981). However, when Cronbach’s alpha value is above 0.70, AVE values greater than 0.40 are acceptable, indicating good convergent validity (Hair et al., 2011).

Discriminant validity was tested by comparing the square root of the AVE values with the correlation values between factors. The square root values of the AVE should be higher than the correlation value between the factors whose discriminant validity is to be analysed.

The sensitivity of the items was tested with all the participants. The items must have answers at all the response points, the median must not be close to one of the extremes, and the absolute values of asymmetry and kurtosis must be below 3 and 7, respectively (Kline, 2011).

Finally, descriptive statistics were carried out for each dimension using one-sample Student’s t tests.

## Results

### Exploratory Factor Analysis

The first step was to test the validity of this instrument through exploratory factor analysis with one of the two parts into which the sample of this study was randomly divided. Exploratory factor analysis aims to discover and analyse the structure of a set of interrelated variables to construct a measurement scale for (intrinsic) factors that (explicitly) control the original variables (Marôco, 2021). A KMO value of 0.91 was obtained, which can be considered very good (Sharma, 1996). Bartlett’s test of sphericity was significant at *p* < 0.001, which is an acceptable value to continue the analysis and an indicator that the data come from a normal multivariate population (Pestana & Gageiro, 2003). This scale’s factor structure was based on six factors, which explained 65% of the scale’s total variability. Item 6 was removed because it had a low factor weight. The remaining items had weights equal to or greater than 0.50, as shown in the table.

This instrument maintained the factor structure proposed by Exline et al. (2014) (Table 2).

**Table 2:** Factors and factor weights of the items obtained in the exploratory factor analysis

	Factor					
	1	2	3	4	5	6
RSS1—I felt guilty for not living up to my moral standards/ Senti-me culpado por não viver de acordo com os meus padrões morais	0.13	0.11	0.11	0.00	0.18	**0.84**
RSS2—I felt angry with God/ Senti-me zangado com Deus	0.03	**0.63**	0.25	0.04	0.20	0.19
RSS3—I’ve had concerns about whether there is an ultimate purpose to life or existence/Tive preocupações sobre se existe um propósito último para a vida ou existência	0.02	0.11	0.13	0.45	0.**57**	0.06
RSS4—I have felt hurt, mistreated, or offended by religious/spiritual people/Senti-me magoado, maltratado ou ofendido por pessoas religiosas/espirituais	0.12	0.11	**0.79**	0.07	0.20	0.07
RSS5—I struggled to find out what I believe about religion/spirituality/Lutei para descobrir em que realmente acredito sobre a religião/espiritualidade	0.06	− 0.06	0.44	**0.51**	0.20	0.22
RSS6—I felt attacked by the devil or evil spirits/Senti-me atacado pelo diabo ou espíritos malignos	**0.85**	0.06	0.14	0.02	0.11	0.21
RSS7—I asked myself if life really matters/Questionei-me se a vida realmente importa	0.14	0.15	0.20	0.21	**0.73**	0.04
RSS8—I felt torn between what I wanted and knew was morally right/Senti-me dividido entre aquilo que eu queria e o que eu sabia ser moralmente correto	0.17	0.20	0.17	0.31	0.34	**0.53**
RSS9—I questioned God’s love for me/Questionei o amor de Deus por mim	0.12	**0.60**	0.20	0.28	0.28	0.00
RSS10—I’ve had conflicts with other people over religious/spiritual matters/Tive conflitos com outras pessoas sobre assuntos religiosos/espirituais	0.11	0.17	**0.68**	0.21	0.10	0.22
RSS11—I felt like the devil (or an evil spirit) was trying to keep me from the good/ Senti-me como se o diabo (ou um espírito mau) estivesse a tentar afastar-me do bem	**0.78**	0.24	0.13	0.09	0.08	0.31
RSS12—I felt as if my life had no deeper meaning/ Senti-me como se a minha vida não tivesse um significado mais profundo	0.17	0.29	0.11	− 0.07	**0.74**	0.15
RSS13—I felt irritated by organized religion/Senti-me irritado com a religião organizada	− 0.03	0.23	**0.50**	0.22	0.39	0.02
RSS14—I worried about whether my actions were morally or spiritually wrong/Preocupei-me se as minhas ações foram moralmente ou espiritualmente erradas	0.26	0.05	0.12	0.42	0.06	**0.60**
RSS15—I felt confused about my religious and spiritual beliefs/ Senti-me confuso acerca das minhas crenças religiosas e espirituais	0.03	0.28	0.27	**0.59**	0.29	0.15
RSS16—I felt like God was punishing me/Senti-me como se Deus me estivesse a castigar	0.28	**0.67**	0.02	0.06	0.13	0.18
RSS17—I felt rejected or misunderstood by religious/spiritual people/ Senti-me rejeitado ou incompreendido por pessoas religiosas/espirituais	0.18	0.22	**0.77**	0.15	0.12	0.04
RSS18—I worried whether the problems I faced were the devil’s or evil spirits’ work/ Preocupei-me se os problemas que estava a enfrentar seriam trabalho do diabo ou de espíritos maus	**0.81**	0.18	0.17	0.11	0.07	0.03
RSS19—I felt as if God had abandoned me/ Senti-me como se Deus me tivesse abandonado	0.27	**0.76**	0.11	0.16	0.12	0.06
RSS20—I worried about whether my beliefs about religion/spirituality were correct/Preocupei-me sobre se as minhas crenças sobre religião/espiritualidade estariam corretas	0.12	0.21	0.13	**0.78**	0.08	0.16
RSS21—I had trouble following my moral principles/ Tive dificuldades em seguir os meus princípios morais	0.18	0.29	0.20	0.30	− 0.02	**0.65**
RSS22—I asked myself if my life was really going to make a difference in the world/ Questionei-me sobre se a minha vida iria realmente fazer alguma diferença no mundo	0.09	0.09	0.17	0.38	**0.55**	0.23
RSS23—I felt troubled by doubts or questions about religion and spirituality/Senti-me perturbado por dúvidas ou questões sobre religião e espiritualidade	0.13	0.22	0.24	**0.66**	0.27	0.17
RSS24—I felt as if God had let me down/ Senti-me como se Deus me tivesse desiludido	0.13	**0.74**	0.21	0.18	0.07	0.11
RSS25—I felt tormented by the devil or evil spirits/ Senti-me atormentado pelo diabo ou por espíritos malignos	**0.84**	0.24	0.06	0.13	0.11	0.05
RSS26—I felt others considered me less because of my religious/spiritual beliefs/ Senti-me como se outros me considerassem menos por causa das minhas crenças religiosas/espirituais	0.27	0.29	**0.52**	0.20	0.02	0.29

Bold indicates the factor weights of each item in the factor to which they belong

Factor 1—Demonic Struggles; factor 2—Divine Struggles; Factor 3—Spiritual Struggles; Factor 4—Struggles with Doubt; Factor 5—Struggles with ultimate Meaning/Purpose; factor 6—Moral Struggles

### Internal Consistency

Regarding internal consistency, all the dimensions had Cronbach’s alpha values above 0.70, indicating good internal consistency (Table 3).

**Table 3:** Internal consistency of the dimensions

Dimension	Number of items	Cronbach’s Alpha
Demonic struggles	4	0.90
Divine struggles	5	0.82
Interpersonal struggles	5	0.81
Struggles with doubt	4	0.80
Struggles with ultimate Meaning	4	0.76
Moral struggles	4	0.79

### Confirmatory Factor Analysis

The next step was to carry out two confirmatory factor analyses, one factor and six factors. The fit indices obtained in the one-factor confirmatory factor analysis were inadequate (χ^2^/gl = 5.93; CFI = 0.69; GFI = 0.67; TLI = 0.66; RMSR = 0.94; RMSEA = 0.12). The fit indices obtained in the six-factor exploratory factor analysis were adequate (χ^2^/gl = 2.63; CFI = 0.91; GFI = 0.87; TLI = 0.89; RMSR = 0.062; RMSEA = 0.067). These results indicate that the participants in this study perceived this instrument as comprising six factors (Table 4). All the items had factor weights greater than 0.50.

**Table 4:** Dimensions and factor weights of the items obtained in the confirmatory factor analysis

Dimension	Item	Factor weights
Demonic struggles	RSS6—I felt attacked by the devil or evil spirits	0.77
	RSS11—I felt like the devil (or an evil spirit) was trying to keep me from the good	0.79
	RSS18—I worried whether the problems I faced were the devil’s or evil spirits’ work	0.83
	RSS25—I felt tormented by the devil or evil spirits	0.80
Divine struggles	RSS2—I felt angry with God	0.55
	RSS9—I questioned God’s love for me	0.77
	RSS16—I felt like God was punishing me	0.79
	RSS19—I felt as if God had abandoned me	0.83
	RSS24—I felt as if God had let me down	0.75
Interpersonal struggles	RSS4—I have felt hurt, mistreated, or offended by religious/spiritual people	0.74
	RSS10—I’ve had conflicts with other people over religious/spiritual matters	0.56
	RSS13—I felt irritated by organized religion	0.66
	RSS17—I felt rejected or misunderstood by religious/spiritual people	0.77
	RSS26—I felt others considered me less because of my religious/spiritual beliefs	0.74
Struggles with doubt	RSS5—I struggled to find out what I believe about religion/spirituality	0.63
	RSS15—I felt confused about my religious and spiritual beliefs	0.76
	RSS20—I worried about whether my beliefs about religion/spirituality were correct	0.73
	RSS23—I felt troubled by doubts or questions about religion and spirituality	0.78
Struggles with ultimate meaning	RSS3—I’ve had concerns about whether there is an ultimate purpose to life or existence	0.63
	RSS7—I asked myself if life really matters	0.73
	RSS12—I felt as if my life had no deeper meaning	0.74
	RSS22—I asked myself if my life was really going to make a difference in the world	0.70
Moral struggles	RSS1—I felt guilty for not living up to my moral standards	0.51
	RSS8—I felt torn between what I wanted and knew was morally right	0.68
	RSS14—I worried about whether my actions were morally or spiritually wrong	0.65
	RSS21—I had trouble following my moral principles	0.75

### Construct Reliability

Concerning construct reliability, all the dimensions had values above 0.70, indicating good construct reliability. Table 5 shows the construct reliability value for each of the dimensions.

**Table 5:** Construct reliability of each dimension

Dimension	Number of items	CR
Demonic struggles	4	0.87
Divine struggles	5	0.86
Interpersonal struggles	5	0.82
Struggles with doubt	4	0.81
Struggles with ultimate Meaning	4	0.79
Moral struggles	4	0.74

### Convergent Validity

Only some dimensions showed values above 0.50 regarding convergent validity, which, according to Fornell and Larcker (1981), indicates good convergent validity. The Demonic Struggles, Divine Struggles and Struggles with Doubt dimensions had values above 0.50 (Table 6). The Interpersonal Struggles and Struggles with Ultimate Meaning dimensions were slightly below 0.50 (Table 6). Only the Moral Struggles dimension had lower AVE values (Table 6). However, as the Cronbach’s alpha value for this dimension was above 0.70, it could also be considered to have good convergent validity (Hair et al., 2011).

**Table 6:** Convergent validity of each dimension

Dimension	Number of items	AVE
Demonic struggles	4	0.63
Divine struggles	5	0.55
Interpersonal struggles	5	0.47
Struggles with doubt	4	0.52
Struggles with ultimate Meaning	4	0.49
Moral struggles	4	0.43

### Discriminant Validity

As shown in Table 7, all square root values of the AVEs were higher than the correlations between the factors for which discriminant validity was tested, ensuring discriminant validity.

**Table 7:** Discriminant validity of each dimension

		1	2	3	4	5	6
1. Divine struggles		**0.74**					
2. Demonic struggles		0.49	**0.79**				
3. Struggles with doubt		0.55	0.37	**0.72**			
4. Moral struggles		0.46	0.51	0.61	**0.66**		
5. Struggles with ultimate meaning		0.55	0.37	0.62	0.51	**0.70**	
6. Interpersonal struggles		0.56	0.44	0.63	0.52	0.54	**0.69**

The square root values of the AVE are shown in bold

### Sensitivity of Dimensions and Items

When the normality of the dimensions was tested, it was found that none followed a normal distribution (*p* < 0.05) (Table 8). However, when we analysed the absolute values of asymmetry and kurtosis, they were below 3 and 7, respectively, indicating that they did not grossly violate normality (Kline, 2011) (Table 8). With regard to asymmetry, all the dimensions had positive asymmetry. For kurtosis, the dimensions of Divine Struggles, Demonic Struggles and Interpersonal Struggles had a leptokurtic distribution (> 0) (Table 8). The dimensions of Struggles with Doubt, Moral Struggles and Struggles with Ultimate Meaning had a platykurtic distribution (< 0) (Table 8).

**Table 8:** Tests of normality, skewness, and kurtosis for each dimension

Dimension	Kolmogorov–Smirnov			Skewness	Kurtosis
	Statistic	df	*p*		
Divine struggles	0.19	732	< 0.001	1.50	2.492
Demonic struggles	0.20	732	< 0.001	1.21	0.821
Struggles with doubt	0.08	732	< 0.001	0.39	− 0.414
Moral struggles	0.07	732	< 0.001	0.14	− 0.455
Struggles with ultimate Meaning	0.10	732	< 0.001	0.45	− 0.447
Interpersonal struggles	0.09	732	< 0.001	0.60	0.193

As far as the sensitivity of the items is concerned, all items had responses at all points. Only Items 6, 9, 11, 16, 18, 19, 24, 25 and 26 had a median towards the lower end. The absolute values of skewness and kurtosis were below 3 and 7, respectively, indicating that they did not grossly violate normality (Kline, 2011) (Table 9).

**Table 9:** Descriptive statistics of the scale’s dimensions

Dimension	t	df	*p*	Mean	SD
Divine struggles	– 52.54	731	< 0.001	1.62	0.71
Demonic struggles	– 40.29	731	< 0.001	1.72	0.86
Struggles with doubt	– 23.86	731	< 0.001	2.22	0.88
Moral struggles	– 19.43	731	< 0.001	2.39	0.84
Struggles with ultimate Meaning	– 21.02	731	< 0.001	2.30	0.91
Interpersonal struggles	– 28.11	731	< 0.001	2.15	0.82

### Descriptive Statistics of the Scale’s Dimensions

The position of the answers given by the participants in this study was the subject of descriptive statistics of the scale’s dimensions.

The results indicate that the participants in this study had a low perception of all the dimensions of the scale, as all of their averages were significantly below the scale’s central point (3) (Table 9). Divine struggles and demonic struggles were the dimensions with the lowest values. The dimension with the highest perception was moral struggles (Table 9).

## Discussion

The main objective of this study was to adapt and validate for the Portuguese population the Religious and Spiritual Struggles Scale developed by Exline et al. (2014), which consists of 26 items in six dimensions: divine struggles, demonic struggles, struggles with doubt, moral struggles, struggles with ultimate meaning and interpersonal struggles. The scale was empirically tested by applying it to 732 individuals with various religious affiliations.

Subsequently, exploratory factor analysis and confirmatory factor analysis were performed to validate this scale. The exploratory factor analysis suggested the existence of six factors, as in the study conducted by the authors of this instrument. The items that constituted each of the six factors were the same as in the initial study. A KMO of 0.91 was obtained, which, according to Sharma (1996), can be considered very good. All items had factor weights of 0.50 or higher.

The confirmatory factor analysis confirmed the existence of six factors. The fit indices obtained were adequate. The factor weights of each of the items were higher than 0.50. For convergent validity, only one of the dimensions (moral struggles) had an AVE value well below 0.50, the minimum acceptable value for good convergent validity (Fornell & Larcker, 1981). However, as this dimension had a Cronbach’s alpha value of over 0.70, according to Hair et al. (2011), it could also be considered to have good convergent validity. Discriminant validity between the factors that made up this instrument was also proven since the square root of the AVE values was higher than the correlation values between the factors for which discriminant validity was tested.

Regarding reliability, all the dimensions of this scale had Cronbach’s alpha values above 0.70, ranging from 0.76 (struggles with ultimate meaning and interpersonal struggles) to 0.90 (demonic struggles). For construct reliability, the values were also higher than 0.70, ranging from 0.74 (moral struggles) to 0.87 (demonic struggles).

Concerning the sensitivity of both the dimensions and the items that constitute them they did not grossly violate normality, as their absolute values of asymmetry and kurtosis were below 3 and 7, respectively (Kline, 2011).

The results showed that in all dimensions, individuals scored low, which may be linked to the type of variable measured (i.e., people often tend to underestimate discomfort felt in the religious and spiritual domain) (Abu-Raiya et al., 2010). The experience of a spiritual battle can be complex for individuals and is accompanied by negative emotions such as guilt and shame with possible interference in the way each person assesses the presence and intensity of this phenomenon in self-reports (Exline et al., 2012).

The dimension with the lowest values was divine struggles, probably because individuals consider disillusionment with God a reprehensible feeling or one that could lead to punishment (Exline et al., 2022; Pargament & Exline, 2022). A dimension with lower results was demonic struggles, which is more difficult to understand and more stigmatized, either because of disbelief in the figure of the Devil or evil spirits or because believing individuals are reluctant to think about it (Breuninger et al., 2019; Pargament & Exline, 2022). At the opposite pole, we find the dimension with the highest score, moral struggles, which showed positive and consistent links with high levels of religious involvement. Moral struggles are not exclusive to believers, but religious/spiritual practice involves a set of explicit guidelines about what is correct, honest, upright, and just, so it is expected that this type of focus and concern about personal actions may be more prevalent when assessing the struggles of people of faith (Exline et al., 2022; Pargament & Exline, 2022).

### Study Limitations

This study has some limitations. The main limitation concerns the data collection process and the fact that a self-report questionnaire with closed-ended questions was used, which may have biased the results. However, several methodological and statistical recommendations were followed to reduce the impact of common method variance (Podsakoff et al., 2003).

## Conclusions

The results of the psychometric qualities of this scale show that it can be used in future empirical studies in the psychology of religion and spirituality. It is also a valuable resource for clinical practice, as religious and spiritual struggles are common. This measure can help individuals identify, understand, and overcome sources of religious and spiritual tension in their lives.
